# Co-Expression of Anti-Rotavirus Proteins (Llama VHH Antibody Fragments) in *Lactobacillus:* Development and Functionality of Vectors Containing Two Expression Cassettes in Tandem

**DOI:** 10.1371/journal.pone.0096409

**Published:** 2014-04-29

**Authors:** Gökçe Günaydın, Beatriz Álvarez, Yin Lin, Lennart Hammarström, Harold Marcotte

**Affiliations:** Department of Laboratory Medicine, Division of Clinical Immunology and Transfusion Medicine, Karolinska Institute at Karolinska University Hospital, Stockholm, Sweden; The Ohio State University, United States of America

## Abstract

Rotavirus is an important pediatric pathogen, causing severe diarrhea and being associated with a high mortality rate causing approximately 500 000 deaths annually worldwide. Even though some vaccines are currently available, their efficacy is lower in the developing world, as compared to developed countries. Therefore, alternative or complementary treatment options are needed in the developing countries where the disease burden is the largest. The effect of *Lactobacillus* in promoting health and its use as a vehicle for delivery of protein and antibody fragments was previously shown. In this study, we have developed co-expression vectors enabling *Lactobacillus paracasei* BL23 to produce two VHH fragments against rotavirus (referred to as anti-rotavirus proteins 1 and 3, ARP1 and ARP3) as secreted and/or surface displayed products. ARP1 and ARP3 fragments were successfully co-expressed as shown by Western blot and flow cytometry. In addition, engineered *Lactobacillus* produced VHH antibody fragments were shown to bind to a broad range of rotavirus serotypes (including the human rotavirus strains 69M, Va70, F45, DS1, Wa and ST3 and simian rotavirus strains including RRV and SA11), by flow cytometry and ELISA. Hereby, we have demonstrated for the first time that when RRV was captured by one VHH displayed on the surface of co-expressor *Lactobacillus*, targeting other epitope was possible with another VHH secreted from the same bacterium. Therefore, *Lactobacillus* producing two VHH antibody fragments may potentially serve as treatment against rotavirus with a reduced risk of development of escape mutants. This co-expression and delivery platform can also be used for delivery of VHH fragments against a variety of mucosal pathogens or production of other therapeutic molecules.

## Introduction

Rotavirus, one of the most crucial pediatric pathogens, is transmitted by the fecal-oral route and infects the enterocytes of the upper- and mid- section of the small intestine [1]. Rotaviral diarrhea is associated with an infant mortality rate of approximately 500 000 children annually worldwide, most of which occur in the developing world [2]. The two oral, live-attenuated licensed vaccines currently available have been found to be less effective in middle-low and low income countries (46–76%) as compared to developed countries (73–98%) [3]–[6].

The rotavirus capsid is composed of three protein layers, out of which VP7 and VP4, G and P types of antigens, respectively, forms the outer layer and are used in a binary classification system to define the viral serotypes/genotypes [1], [7]. According to a surveillance report by WHO, during 2010, the common rotavirus G types (G1–G4 and G9) represented approximately 70% of all rotavirus infections [8]. The frequency of G–P combinations vary geographically, for example G1 P[8], the main component of rotavirus vaccines, was identified in more than 80% of all reported rotavirus infections in America, Europe and the West pacific region, but less than 40 and 20% in Africa and Asia, respectively [9]. However, uncommon rotavirus G/P combinations (G12 P[8], G12 P[6], G2 P[6], G3 P[6], and G1 P[6] viruses), with an emerging G12 type [10], were found in 30 and 50% of all rotavirus infections in Africa and Asia, respectively [9]. A successful rotavirus therapy should thus serve as a good heterotypic protection against human rotaviruses with high diversity and capacity of acquiring mutations.

Genetically modified lactobacilli for *in situ* delivery of antibodies or antibody fragments might represent a potential treatment strategy for acute gastroenteritis. Lactobacilli are normal residents of the gastrointestinal tract and contribute to host health. Some recent, controlled, clinical trials have shown that certain strains of lactobacilli may exhibit prophylactic as well as therapeutic properties in the prevention and treatment of rotavirus diarrhea in children in both developed countries and the developing world [11]–[15].

The variable domain of Camilidae heavy chain antibodies (VHH) consists of a single immunoglobulin domain and is known as the smallest functional antigen-binding fragment known to date [16]. In addition to its antigen binding capacity similar to that of complete antibodies, their acid resistance property contributes to long term stability in the harsh gastrointestinal environment [17]. VHH antibody fragments against rotavirus produced in rice or yeast were shown to be protective in a mouse model of rotavirus infection [18], [19]. Furthermore, they have been shown to be expressed in lactobacilli in a functional conformation and at higher levels than single chain antibody fragments [12], [20], [21].

The production of one VHH fragment targeting a single epitope has limitations due to reduced cross-reactivity to circulating viral serotypes and potential development of viral escape mutants, whereas targeting multiple epitopes could increase the efficacy due to a broader specificity towards a range of viral serotypes and a low likelihood of accumulating viral escape mutants.

It has previously been shown that two selected yeast produced VHH antibody fragments (referred to as anti-rotavirus proteins 1 and 3, or ARP1 and ARP3) are directed against different epitopes and able to neutralize a broad range of rotavirus serotypes/genotypes, including recently emerged strains, *in vitro* [22]. The ARP1 and ARP3 fragments were shown to act synergistically against the virus *in vitro* [22] and in a neonatal mouse model of rotavirus-induced diarrhea [12]. Furthermore, *Lactobacillus* producing an anchored ARP3-ARP1 dimer was superior at reducing the rate of diarrhea in a mouse model of rotavirus infection when compared to *Lactobacillus* displaying monovalent ARPs [12]. In this paper, we used these two well-characterized ARP fragments, ARP1 and ARP3, to develop various expression cassettes for co-expression of the two antibody fragments in secreted and cell wall-anchored forms.

## Materials and Methods

### Bacterial Strains, Culture and Plasmids

*E. coli* DH5α (Invitrogen, Carlsbad, CA) was grown in Luria-Bertani (LB) broth at 37°C with 200 rpm orbital shaking or on LB-agar plates at 37°C. *Lactobacillus paracasei* BL23 (previously named *L. casei* ATCC 393 pLZ15^−^) was grown in lactobacilli MRS broth (Difco, Sparks, MD) at 37°C without agitation to an OD_600_ equal to 0.8 (10^8^ cfu/ml) or anaerobically on MRS-agar plates (BD - GasPak EZ, Sparks, MD). The concentrations of erythromycin used were 5 µg/ml for *L. paracasei* BL23 transformants and 300 µg/ml for *E. coli* DH5α transformants. The plasmids used in this study are listed in Table 1.

**Table 1:** Plasmids used in this study.

Plasmids	Products in *L. paracasei*	Reference
pAF100-ARP1	Secreted ARP1 fused to E-tag	[21]
pAF900-ARP1	Surface-anchored ARP1 fused to E-tag	[21]
pAF900-ARP3(VSV)	Surface-anchored ARP1 fused to VSV-G tag	This study
pAF900-ARP3(HA)	Surface-anchored ARP3 fused to HA-tag	This study
pAF900-ARP3(FLAG)	Surface-anchored ARP3 fused to FLAG-tag	This study
pAF900-ARP3(V5)	Surface-anchored ARP3 fused to V5-tag	This study
pAF1200	Double expression cassette fusion: SecretedARP1 fused to E-tag, and secreted ARP3fused to V5-tag	This study
pAF1300	Double expression cassette fusion: SecretedARP1 fused to E-tag, and surface-anchored ARP3fused to V5-tag	This study
pAF1400	Double expression cassette fusion: Surface-anchored ARP1 fused to E-tag, and surface-anchoredARP3 fused to V5-tag	This study

### Virus Stock Preparations

The human rotavirus strains (69M (G8P[10]), Va70 (G4P[8]), F45 (G4P[8]), DS-1 (G2P[4]), Wa (G1P[8]) and ST3(G4P[6]), and the simian rotavirus strains (RRV (G3P[3]) and SA11 (G3P[1])) were grown and purified as mentioned earlier [23], [24].

### Generation of Anti-rotavirus Antibody Fragments: ARP1 and ARP3

Immunization of Ilamas with rhesus rotavirus (RRV; strain MMU18006, P5B, G3), followed by selection of ARP1 and ARP3 has been previously described [19], [22]. ARP1 was previously referred to as 2B10 [19] or VHH1 [20]. ARP1 and ARP3 produced in *Saccharomyces cerevisae*, were purified using ion exchange chromatography by BAC BV (The Netherlands). The purity was >95%.

### Construction of Single ARP1 and ARP3 Expression Cassettes and Selection of the Tag

The ARP1 antibody fragment encoding gene was previously cloned in the pAF100 and pAF900 plasmids, resulting in pAF100-ARP1 and pAF900-ARP1, which mediate secretion and surface display of ARPs, respectively [21] (Table 1). In these plasmids, the transcription of the ARPs is regulated by the constitutive promoter of the *aggregation promoting factor (apf)* gene from *Lactobacillus crispatus* M247 and secretion outside the cell is mediated by the signal peptide of the *apf* gene. In pAF900, the ARP encoding gene is fused to the sequence encoding the last 231 amino acids of the proteinase P protein (PrtP) of *L. paracasei* BL23 for covalent surface binding of the ARP fragment on the cell wall. ARP1 gene was also fused to the gene encoding an E-tag (GAPVPYPDPLEPR) for detection with an anti-E-tag antibody. A similar expression system was used in the present study.

**Figure 1:**
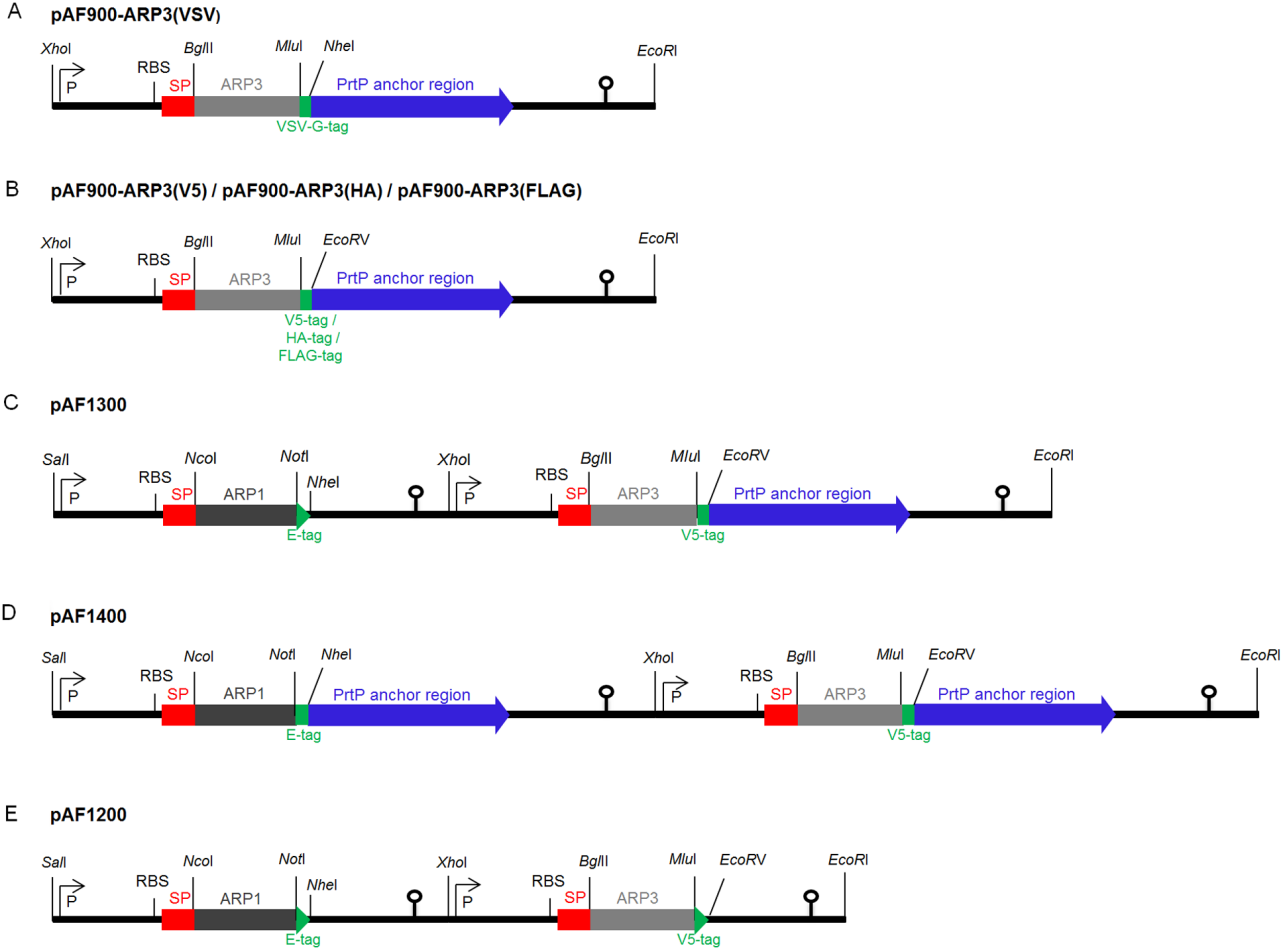
Single expression cassettes for surface display of ARP3 fused to VSV-G (A); and V5/HA/FLAG-tag (B); and co-expression cassettes where one ARP fragment can be secreted (ARP1) and the other (ARP3) anchored on the cell surface (C); both covalently anchored on the cell surface (D); or both secreted in the medium (E). SP, signal peptide; RBS, ribosomal binding site; P, *apf* promoter; translational stop codon indicated with an arrowhead and the transcription terminator indicated with a lollipop.

Before construction of the lactobacilli co-expressing ARP1 and ARP3, another tag was selected for fusion to ARP3 in order to detect each antibody fragment separately. The gene encoding the sequence of ARP3, VSV-G-tag, *prtP* and transcription terminator of the *apf* gene was synthesized (Genscript, Piscataway, NJ). During the synthesis, restriction sites were added for subsequent fusion of the two expression cassettes and replacement of VHH genes. Furthermore, the sequence was optimized according to the codon usage of *L. paracasei* in order to reduce potential recombination between homologous DNA sequences in the ARP1/ARP3 double expression cassettes. The synthetic gene was subsequently fused to the *apf* promoter and cloned between the *EcoR*I and *Pvu*I restriction enzyme sites in the pIAV7 plasmid [25], generating pAF900-ARP3(VSV) (Fig. 1 A). Subsequently, the VSV-G-tag in pAF900-ARP3(VSV) was replaced with the codon adjusted nucleotide sequences of the V5- (GKPIPNPLLGLDST), HA- (YPYDVPDYA) and FLAG- (DYKDDDDK) tags generating pAF900-ARP3(FLAG), pAF900-ARP3(HA) or pAF900-ARP3(V5) plasmids, respectively (Fig. 1 B). The construction of the pAF900 plasmids is described in detail in Materials and Methods S1.

### Construction of Two Fused ARP1 and ARP3 Co-expression Cassettes

For co-expression of ARP1 and ARP3 by lactobacilli, the expression cassettes producing ARP1 and ARP3 were fused. The expression cassettes of pAF100-ARP1, and pAF900-ARP1 were cut using *Sal*I and *EcoR*I restriction enzymes and separately ligated into similarly digested pAF900-ARP3(VSV) vectors, generating pAF1300-VSV or pAF1400-VSV plasmids, respectively (figure not shown). The VSV-G-tag encoding sequences were subsequently replaced by the V5-tag sequences in both of these vectors as it improved detection of the ARP3 gene, generating pAF1300 (Fig. 1 C) and pAF1400 (Fig. 1 D), respectively (see Material and Methods S1). A distinct cloning strategy was used to generate pAF1200 mediating secretion of both ARPs. The *apf* terminator region was amplified from the plasmid pAF100-ARP1 using TR12_Fw_SVHH3 and TR13_Rv_SVHH3 primers (Table S1), introducing a *Mlu*I site, a V5-tag sequence, a stop codon and an *EcoR*V site upstream of the terminator region and replacing the *EcoR*I to a *Pvu*I site downstream of the terminator region. The amplicon was digested with *Mlu*I and *Pvu*I and inserted between the same restriction sites into the plasmid pAF1300, generating pAF1200 (Fig. 1 E).

The plasmids were transformed into *L. paracasei* BL23 by electroporation as described previously [26].

### Western Blot Analysis

Expression of ARP1 and ARP3 produced by the transformed lactobacilli was analyzed by Western blot. The preparation of supernatants and cell extracts as well as gel electrophoresis and blotting conditions have been described elsewhere [27].

The expression of ARP1, fused to an E-tag, was detected by a mouse monoclonal anti-E-tag antibody (Phadia AB, Uppsala, Sweden, 1 µg/ml), followed by a HRP conjugated goat anti-mouse antibody (Dako A/S, Glostrup Denmark, 1∶1000). The expression of the ARP3 gene fused to different commercial tags, including VSV-, HA-, FLAG- and V5-tags, were evaluated by various primary and secondary antibodies in Western blot (see Table S2), and the level of detection was indicated by “−” (no detection), “+” (low detection) or “++” (high detection). ARP3 fused to V5-tag was detected successfully using a biotinylated mouse monoclonal anti-V5-tag antibody (AbD Serotec, Kidlington, UK, 1∶5000), followed by a HRP conjugated streptavidin (Becton, Dickinson and Company, Franklin Lakes, NJ, USA, 1∶1000).

Furthermore, the amount of ARP1 and ARP3 fragments produced in the supernatant and cell extract of *Lactobacillus* cultures was quantified by Western blot densitometry as compared to known concentrations of affinity purified ARP1 and ARP3 proteins [21], [26]. The VHH were purified from supernatant from *L. paracasei* pAF1200 using HiTrap sepharose (GE-healthcare) coupled to anti-V5 or anti-E-tag monoclonal antibodies according to the manufacturer’s instructions. The amount of expressed VHH in the supernatant was given in µg/ml while the amount in the cell extract was calculated as molecule/bacteria based on the molecular weight of the VHH fragments and the number of bacteria in the culture.

### Flow Cytometry Analysis

To assess the display of ARP1, bacteria (2×10^7^ cfu) were incubated with an anti-E-tag antibody (1∶100) followed by a fluorescein isothiocyanate (FITC) conjugated goat anti-mouse antibody (Jackson Immunoresearch Lab., West Grove, PA, USA, 1∶200). For detection of ARP3 fused to the V5-tag, a biotinylated mouse monoclonal anti-V5-tag antibody (1∶400) followed by FITC conjugated streptavidin (Biolegend, San Diego, CA, 1∶200) were used. All antibody incubations took place on ice for 30 min, and the cells were then washed with chilled PBS. Prior to analysis, the cells were fixed by 0.5% paraformaldehyde (PFA) for 15 min. A FACS Calibur machine (Becton, Dickinson and Company) was used for cell staining analysis.

Subsequently, the binding of lactobacilli producing surface-anchored VHH antibody fragments to human (69M, Va70, F45, DS1, Wa and ST3) and simian rotavirus strains (RRV and SA11) was assessed by flow cytometry. Bacteria were incubated separately with each rotavirus strain (1,5×10^3^ ffu) on ice for 30 min and washed twice with PBS. The virus was detected using a broadly cross-reactive biotinylated polyclonal rotavirus specific antibody derived from hyperimmune bovine colostrum (HBC) (5 µg/ml) [28] and phycoerythrin (PE) conjugated streptavidin (Biolegend, San Diego, CA, 1∶200).

Simultaneous binding of rotavirus by surface-anchored ARP3 and secreted ARP1 produced by *L. paracasei* pAF1300 was also evaluated. The transformed bacteria (2×10^7^ cfu, 200 µl) were mixed to RRV (20 µl) containing MRS medium and grown for 30 min or 1 h at 37°C. The cells were centrifuged and collected for detection of ARP1 and rotavirus bound on the surface of the lactobacilli by flow cytometry, as described previously. *L. paracasei* pAF900-ARP3(V5) was also incubated in MRS medium containing rotavirus and 100 ng of purified ARP1 equivalent to the amount of ARP1 in the culture supernatant. The results were compared to a mixture of *L. paracasei* pAF900-ARP3(V5) producing surface-anchored ARP3 and *L. paracasei* pAF100-ARP1 secreting ARP1 (2×10^7^ cfu of each strain in 200 µl) similarly incubated with rotavirus. Non-transformed *L. paracasei* BL23 was used as a negative control and *L. paracasei* pAF900-ARP1 was used as a positive control for ARP1 staining.

### Enzyme Linked Immunosorbent Assay (ELISA)

The binding of ARP fragments secreted in the supernatant of lactobacilli transformed with pAF100-ARP1, pAF1200 and pAF1300 was determined by ELISA. Ninety-six well ELISA plates were coated with capturing anti-rotavirus HBC antibodies (5 µg/ml), for 2 h at room temperature, prior overnight coating with rotaviruses (69M, Va70, F45 and RRV) at 4°C. Upon blocking with 5% milk, two-fold serial dilutions of supernatants (starting from a 1∶10 dilution) were incubated at 4°C overnight. ARP1 was detected using a mouse anti-E-tag antibody (1∶2000) followed by an alkaline phosphatase (AP) conjugated rabbit anti-mouse antibody (Dako, 1∶1000), and ARP3 was detected using a biotinylated anti-V5-tag antibody followed by AP conjugated streptavidin (Becton, Dickinson and Company, 1∶1000). ELISA was developed with 1 mg/ml of p-nitrophenyl phosphate (Sigma-Aldrich) in 1 M diethanolamine buffer (pH 9.8) followed by optical density measurement at 405 nm. The supernatant of non-transformed lactobacilli was used as negative control.

## Results and Discussion

Modified lactobacilli producing VHH antibody fragments offer a novel approach to the prevention and treatment of rotavirus-induced diarrhea and could complement current vaccine-based forms of prophylaxis. Engineered lactobacilli producing surface-anchored ARP1 and ARP3 were previously generated and shown to be effective in a mouse pup rotavirus infection model [12], [20].

The development of single expression cassettes for expression of antibody fragments fused to an E-tag encoding sequence was previously published [21]. The expression cassette in pAF100 plasmid mediates secretion of antibody fragments from *Lactobacillus* in the supernatant, whereas the pAF900 plasmid mediates covalent anchoring of ARP fragments on the cell surface. In this study, we have developed vectors consisting two expression cassettes cloned in tandem to allow the co-expression of the two functional ARP antibody fragments, thus targeting different epitopes simultaneously.

### Selection of Tag for Detection of ARP3

To distinguish the co-produced ARP1 and ARP3 by *Lactobacillus*, an additional tag was selected among various available commercial ones (VSV-G, V5, HA and FLAG) for fusion to ARP3. These tags are distinct in length, flexibility, hydrophobicity and net charge of their amino acid sequence.

Antibodies raised against the four aforementioned tags were tested in Western blot using the cell extracts of *L. paracasei* pAF900-ARP3(VSV), pAF900-ARP3(V5), pAF900-ARP3(HA) and pAF900-ARP3(FLAG) (Table S2). Surface-anchored ARP3 fused to VSV-, HA- or FLAG-tag could not be detected using a mouse monoclonal anti-VSV or rabbit polyclonal anti-HA and anti-FLAG-tag antibodies, respectively (Table S2). A faint band of the appropriate size was observed in *L. paracasei* pAF900-ARP3(V5) when detected by a mouse monoclonal anti-V5-tag antibody. The difficulty in detecting some of the tags could be due to the folding of the ARP3-tag fusion protein, hindering the epitope or to the quality of the commercial antibody preparation tested. Subsequently, by using a biotinylated mouse monoclonal anti-V5-tag antibody, ARP3 fusion protein was detected with a strong signal in the cell extract of *L. paracasei* pAF900-ARP3(V5). Therefore, we decided to fuse ARP3 to V5-tag for further construction of the double expression cassette and to detect ARP3-V5-tag using the same antibody.

### Construction of Lactobacilli Co-expressing Two Antibody Fragments, ARP1 and ARP3

Three different double expression cassettes were constructed where one ARP fragment is secreted (ARP1) and the other one (ARP3) is anchored on the cell surface, pAF1300 (Fig. 1 C); both covalently anchored on the cell surface, pAF1400 (Fig. 1 D); or both secreted in the medium, pAF1200 (Fig. 1 E). In these cassettes, the gene encoding the ARP1 fragment is fused to an E-tag while the gene encoding the ARP3 fragment is fused to a V5-tag (Table 1). Different restriction sites flanking ARP1 (*Nco*I and *Not*I) and ARP3 (*Bgl*II and *Mlu*I) were introduced to facilitate their replacement by other VHH fragments encoding genes in the future (Fig. 1 C–E).

**Figure 2:**
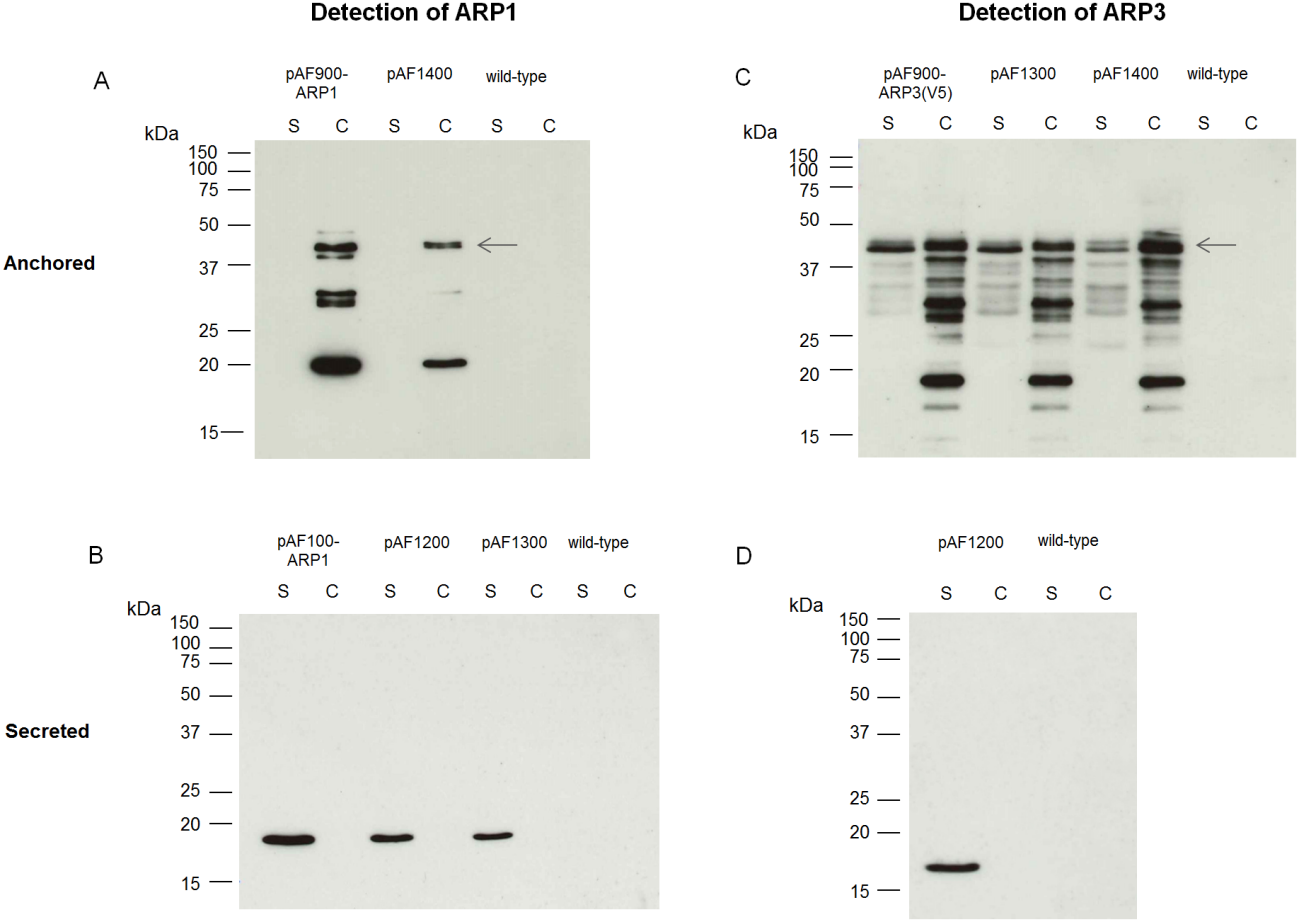
Expression of ARP1 and ARP3 by transformed *L. paracasei* BL23 in Western blot. Expression of surface-anchored (A) and secreted (B) ARP1, and surface-anchored (C) and secreted (D) ARP3 by *L. paracasei* BL23 transformed with pAF100, pAF900, pAF1200, pAF1300 and pAF1400 vectors was analyzed in supernatant and cell extracts. S, supernatant; C, cell extract.

ARP1 and ARP3 fragments were successfully co-expressed from the double expression cassettes as shown by Western blot (Fig. 2). The ARP1 anchored fusion protein (45 kDa) was detected in the cell extract of *L. paracasei* pAF900-ARP1 and *L. paracasei* pAF1400 (Fig. 2 A), whereas secreted ARP1 (21 kDa) was detected in the supernatant of *L. paracasei* pAF1300 and *L. paracasei* pAF1200 (Fig. 2 B). Similarly, ARP3 anchored fusion protein (46 kDa) was detected in the cell extract of *L. paracasei* pAF900-ARP3(V5) and *L. paracasei* pAF1400 (Fig. 2 C), whereas the supernatant of *L. paracasei* pAF1200 revealed a band of 21 kDa, migrating at the expected size of the secreted ARP3 (Fig. 2 D). The level of expression as determined by Western blot densitometry was similar between lactobacilli co-expressing the two ARPs and lactobacilli producing one fragment. The concentration of VHH secreted in the supernatant varied between 1 and 2 µg/ml while the number of VHH anchored on the cell surface varied between 2×10^3^−6×10^3^ molecules/bacteria (Table 2).

**Table 2:** Amount of ARP1 and ARP3 in transformed *Lactobacillus* BL23 producing one or two ARPs.

	Number of ARP molecules per bacterium		Concentration of ARP secreted in supernatant (µg/ml)	
Plasmid	ARP1	ARP3	ARP1	ARP3
	(anchored)	(anchored)	(secreted)	(secreted)
pAF900-ARP1	3×10^3^	-	-	-
pAF900-ARP3(V5)	-	5×10^3^	-	-
pAF1200	-	-	1.2	2.2
pAF1300	-	3×10^3^	1.2	-
pAF1400	2×10^3^	6×10^3^	-	-

**Figure 3:**
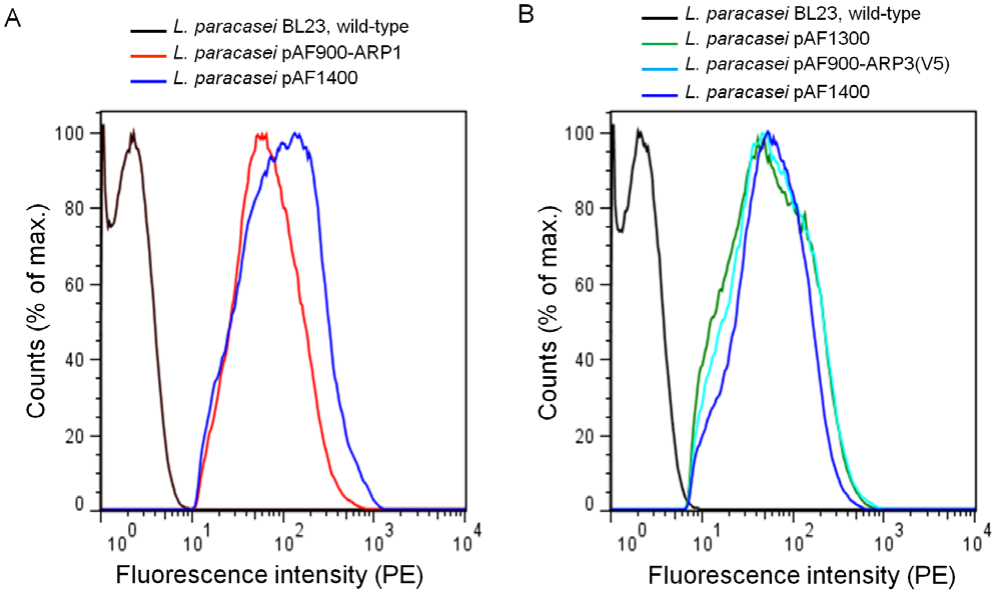
Surface display of ARP1 and ARP3 by transformed *L. paracasei* BL23 in flow cytometry. Surface display of anchored ARP1 (A) and ARP3 (B) produced by *L. paracasei* BL23 transformed with vectors mediating single and co-expression of ARPs as compared to non-transformed wild-type *L. paracasei* BL23.

The display of anchored ARP1 (Fig. 3 A) and ARP3 (Fig. 3 B) fragments on the surface of engineered *L. paracasei* BL23 cells was determined by flow cytometry. Both ARP fragments were displayed in equal amounts on the surface of lactobacilli producing one or two fragments. The median of fluorescence intensities were approximately 100-fold increased for the engineered lactobacilli producing ARP1 and ARP3 as compared to a non-expressor wild-type *L. paracasei* BL23 strain (Fig. 3 A, B).

### Assessment of the Binding of ARPs to Various Rotavirus Strains

**Figure 4:**
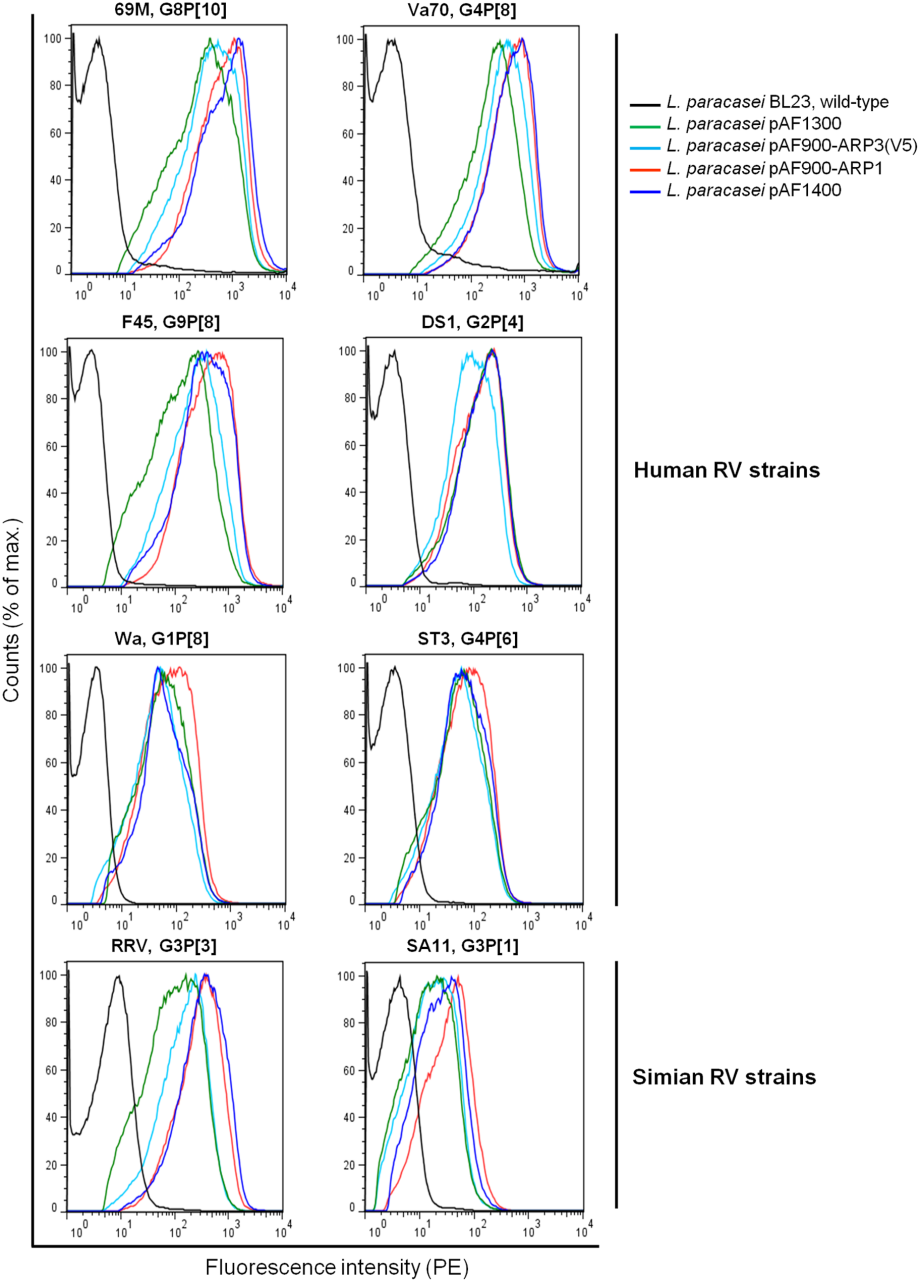
Binding activity of anchored ARP1 and ARP3 produced by transformed *L. paracasei* BL23 cells to human and simian rotavirus strains with distinct genotypes in flow cytometry.

Lactobacilli producing surface-anchored ARPs were tested for binding to a broad range of different rotavirus genotypes, representative of the most common strains worldwide. The ARP single expressor and co-expressor lactobacilli were found to be equally effective in binding to all rotavirus strains by flow cytometry (Fig. 4). Lactobacilli producing surface-anchored ARPs were shown to bind to all the strains although the fluorescence intensity varied. The median fluorescence intensity was superior for 69M, Va70 and F45 human rotavirus strains (100–1000 fold more), followed by DS1, Wa, RRV and ST3 (100-fold more) and lastly SA11 (10-fold more), as compared to the negative control, wild-type *L. paracasei* BL23. ARPs might bind with different affinity to various strains but this difference could also be due to virus preparations including different ratio of double and triple layered viral particles or distinct binding affinity of HBC antibodies use for detection of rotavirus strain. However, the results of binding capacity are in accordance with previous results, where yeast produced ARP1 and ARP3 fragments were shown to neutralize F45 and Va70 more efficiently than ST-3 and DS1, while neither of the fragments were able to neutralize SA11 in the Caco-2 cell line [22].

**Figure 5:**
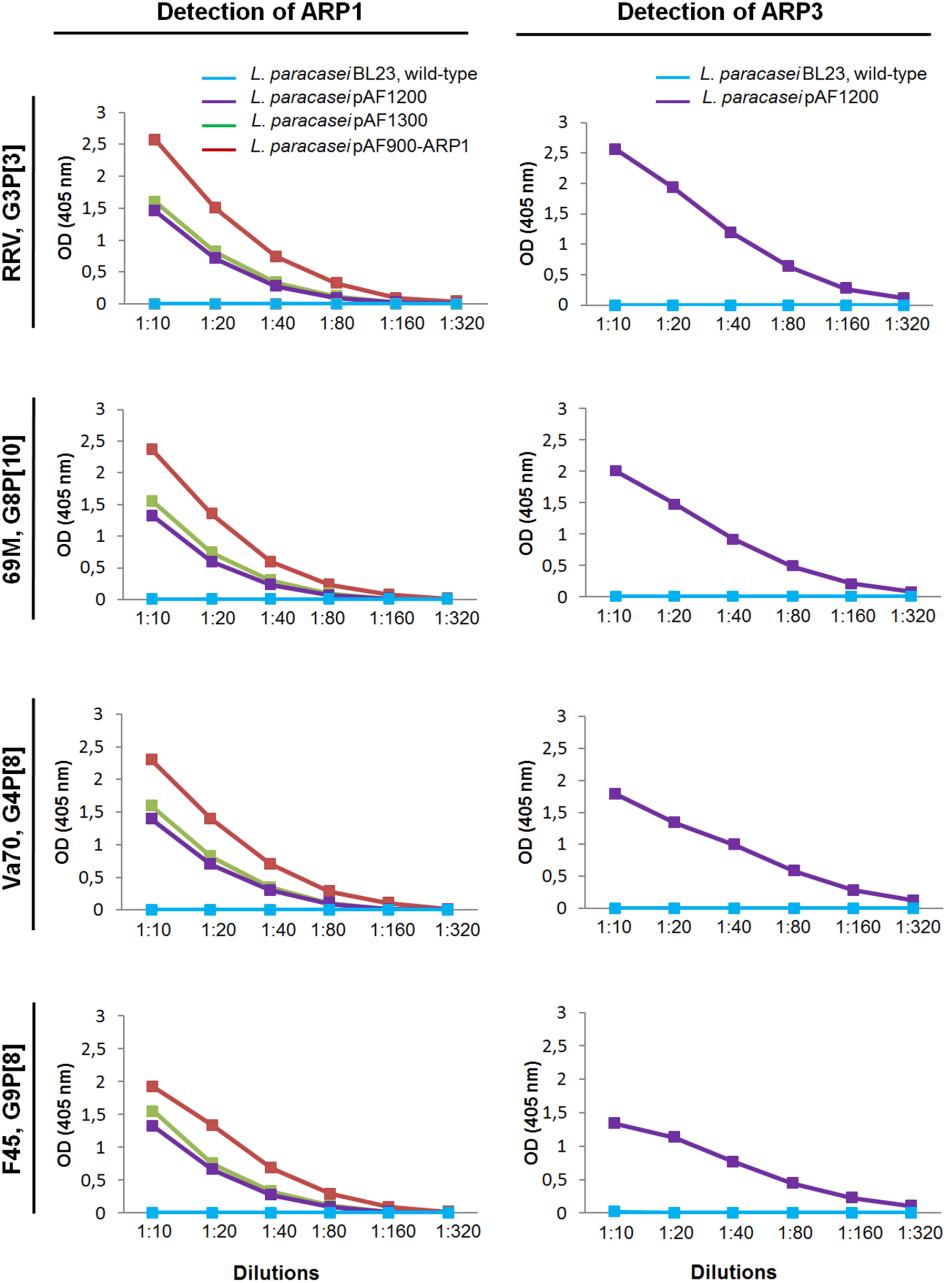
Binding activity of culture supernatant from transformed *L. paracasei* BL23 producing secreted ARP1 and ARP3 to human and simian rotavirus strains with distinct genotypes in ELISA. The binding of secreted ARP1 (A) and ARP3 (B) produced in the supernatant of *L. paracasei* BL23 transformed with pAF100, pAF1200 and pAF1300 vectors was compared to non-transformed wild-type *L. paracasei* BL23.

The functionality of ARP1 and ARP3 fragments, secreted in the cultures supernatant, was evaluated by ELISA (Fig. 5). For this experiment, four rotavirus strains (69M, Va70, F45 and RRV) were selected according to the results of flow cytometry experiments. The binding activity of ARP1 secreted in the supernatant of *L. paracasei* pAF100-ARP1 was approximately two-fold higher than that in the supernatant of lactobacilli transformed with pAF1200 and pAF1300. In addition, the binding activity of ARP3 secreted in the supernatant of *L. paracasei* pAF1200 was approximately two-fold higher than ARP1 secreted in the supernatant of *L. paracasei* pAF1200 (Fig. 5). Thus, ARP1 and ARP3 produced by engineered lactobacilli are broadly cross-reactive to rotaviruses with G1–G4, G8 and G9 serotypes. Although ARPs producing lactobacilli were previously shown to bind and neutralize simian rotavirus RRV [12], [20], this study shows, for the first time, that engineered *Lactobacillus* is able to bind to human rotavirus strains with a high affinity.

### Simultaneous Binding of Anchored ARP3 and Secreted ARP1 to Rotavirus Experiment *In vitro*

**Figure 6:**
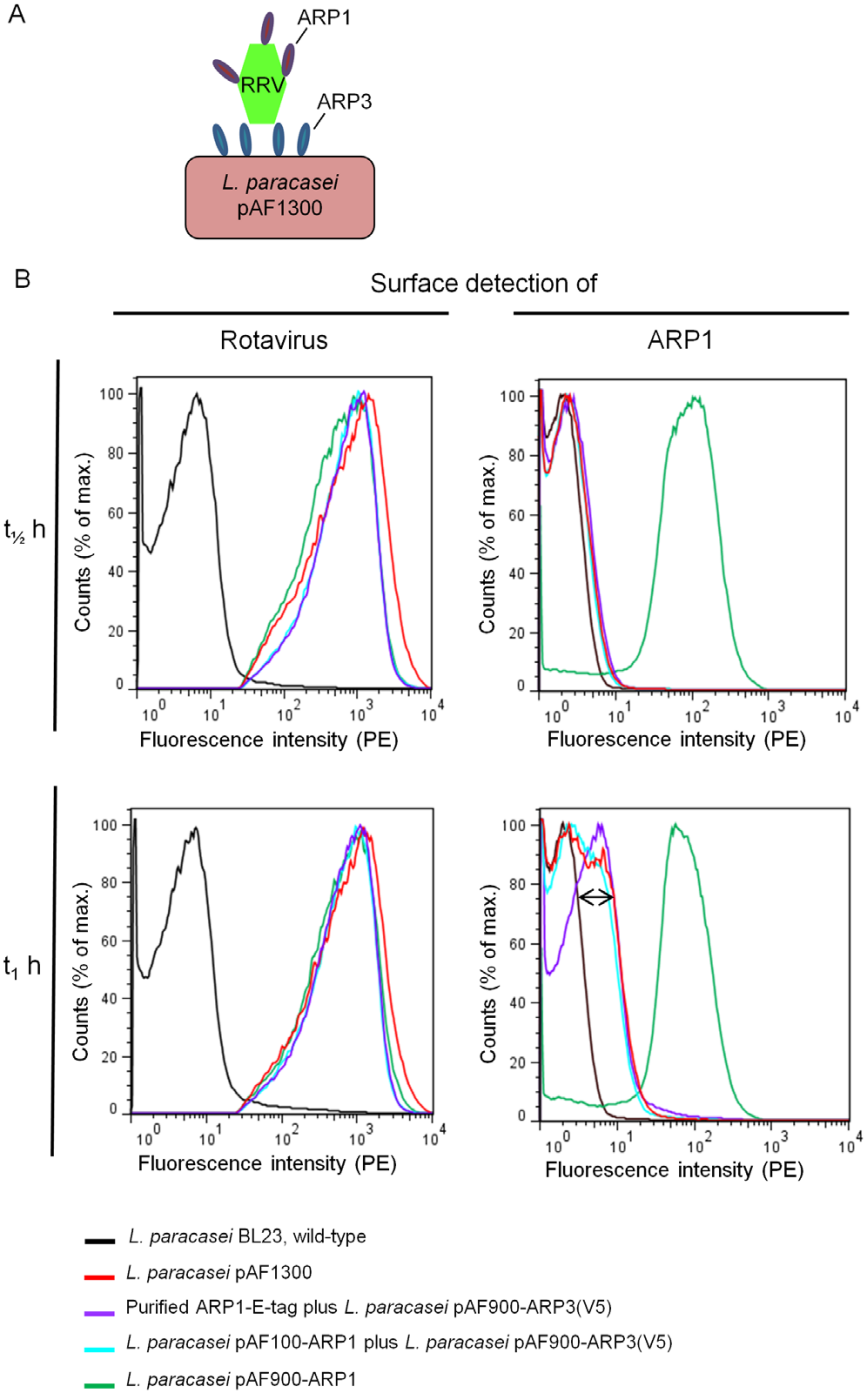
Simultaneous binding experiment: A binding scenario *in situ*. Following incubation of *L. paracasei* pAF1300 with rotavirus (RRV) in MRS medium for 30 min and 1 hour, RRV bound on the surface of modified lactobacilli was detected using biotinylated HBC anti-rotavirus antibodies and PE conjugated streptavidin, and secreted ARP1 bound on the surface of rotavirus was detected using a mouse monoclonal anti-E tag antibody, and FITC conjugated anti-mouse antibodies. The double-headed arrow indicates the positive shift in fluorescence intensity.

Different mechanism(s) might be engaged in binding to rotaviruses when the fragments are co-expressed in *L. paracasei* pAF1300, producing surface-anchored ARP3 and secreted ARP1. The anchored ARP3 displayed on the surface of *Lactobacillus* might initially capture rotavirus particles which may be followed by ARP1 binding to its epitopes on stabilized rotavirus, or secreted ARP1 fragment might bind rotavirus first, followed by binding by ARP3 anchored on the surface of the bacteria (Fig. 6 A).

To mimic the *in situ* mechanism of the interaction of co-expressed ARP1 and ARP3 fragments with rotavirus, *L. paracasei* pAF1300 was incubated with RRV for 30 and 60 min in MRS culture medium and the lactobacilli were subsequently stained both for the presence of RRV and ARP1. After a 30 min incubation (t_1/2 _hr), RRV was detected on the surface of lactobacilli indicating binding of surface-anchored ARP3 to the virus but ARP1 was not detected (Fig. 6 B). Following an additional 30 min incubation period (t_1 _hr), a positive shift for anti-mouse FITC stained bacterial population, corresponding to a 10-fold increased binding activity as compared to the negative control, was observed as an indication of binding of secreted ARP1 on the cell surface bound rotavirus (Fig. 6 B). Furthermore, the binding of ARP1 secreted in the supernatant to RRV particles captured by anchored ARP3, was found to be similar to ARP1 protein purified from the supernatant of modified *Lactobacillus* added in equal amounts (Fig. 6 B). This experiment suggests that once the viral particle is captured by the engineered bacteria, targeting other epitopes with secreted VHH is possible. Even though there was no difference in binding of ARP1 to rotavirus when using co-expressor *Lactobacillus* compared to a mix of *L. paracasei* pAF100-ARP1 secreting ARP1 and *L. paracasei* pAF900-ARP3(V5) producing surface-anchored ARP3 *in vitro* (Fig. 6 B), co-expression of two VHH antibody fragment would decrease the cost of production since there is no need of growing distinct batches of single-expressor bacterial cultures.

## Conclusion

We have previously successfully engineered lactobacilli into producing ARP1 and ARP3 dimers [12]. The currently described system also allows the simultaneous production of two different secreted and/or anchored ARPs. Furthermore, two VHH heterodimers could theoretically be expressed in the future, allowing the production of four VHH antibodies. Expression of distinct rotavirus-specific fragments in *Lactobacillus* could increase the chance of neutralization through binding to different epitopes of the virus. Furthermore, if rotavirus acquires mutations in a certain epitope to escape neutralization, the other ARP will keep their reactivity against the virus. The expressed genes were regulated by two separate promoters instead of one in this plasmid based expression system to avoid any changes in the level of transcription of the downstream gene, the transcription dynamics or stability. In addition, by co-expressing the ARP antibody fragments in one vector, bacteria produce both of them in similar amounts, resulting in a reduced production costs in comparison to a mix of single-expressors. A biologically safe and contained expression system in which the double expression cassettes would be integrated on the chromosome of lactobacilli and allowing transformed lactobacilli to be administered to humans has previously been developed in our laboratory [21]. Modified lactobacilli co-expressing antibody fragments could complement vaccination for treatment of rotavirus infection. This co-expression cassette based platform could also potentially be used for co-expression of VHH fragments targeting other mucosal pathogens or for co-expression of other therapeutic proteins.

## Supporting Information

Table S1 **Primers used in the construction of single or co-expression cassettes.**
(DOCX)

Table S2 **Antibodies used in Western blot for detection of ARP1 and ARP3.**
(DOCX)

Materials and Methods S1 (DOCX)
