# Supplementary material for: Co-Expression of Anti-Rotavirus Proteins (Llama VHH Antibody Fragments) in Lactobacillus: Development and Functionality of Vectors Containing Two Expression Cassettes in Tandem
Source: PLoS One. 2014 Apr 29;9(4):e96409. doi: 10.1371/journal.pone.0096409 (PMC4004553; doi:10.1371/journal.pone.0096409)
Supplement: Table S1 — Primers used in the construction of single or co-expression cassettes. (DOCX) [file pone.0096409.s001.docx]

**Table S1:** Primers used in the construction of single or co-expression cassettes.

| Primer name | Sequence (5´ to 3´) | Features |
| --- | --- | --- |
| TR_5_F | attctcgagggataaggcagaataatgg | Forward, *Xho*I |
| TR_6_R | aatgagatctccggctgggccgcagatccttcta | Reverse, *Bgl*II |
| TR_4 | agctgaattccgatcgggatcccttgaaccgtttg | Reverse, *EcoR*I, *Pvu*I, *BamH*I |
| Fw_MluI_FLAG | gtgacgcgtgattataaagatgatgatgataaagatatcaagaaaacaagtttgttaaa | Forward, *Mlu*I, FLAG-tag, *EcoR*V |
| Fw_MluI_HA | gtgacgcgttatccttatgatgttccagattatgctgatatcaagaaaacaagtttgttaaa | Forward, *Mlu*I, HA-tag*, EcoR*V |
| Fw_MluI_V5 | gtgacgcgtggtaaaccaattccaaatccactactaggcctagatagtaccgatatcaagaaaacaagtttgttaaa | Forward, *Mlu*I, V5-tag, *EcoR*V |
| TR-12_Fw_SVHH3 | cgacgcgtggtaaaccaattccaaatccactactaggcctagatagtaccgatatctaagctagctcaactgtaagtggttcagaa | Forward, *Mlu*I, V5-tag, *EcoR*V |
| TR-13_Rv_SVHH3 | cgatcgcttgaaccgtttgtggtgtcgtttcgtgtatta | Reverse, *Pvu*I |
