# Supplementary material for: Co-Expression of Anti-Rotavirus Proteins (Llama VHH Antibody Fragments) in Lactobacillus: Development and Functionality of Vectors Containing Two Expression Cassettes in Tandem
Source: PLoS One. 2014 Apr 29;9(4):e96409. doi: 10.1371/journal.pone.0096409 (PMC4004553; doi:10.1371/journal.pone.0096409)
Supplement: Table S2 — Antibodies used in Western blot for detection of ARP1 and ARP3. (DOCX) [file pone.0096409.s002.docx]

**Table S2:** Antibodies used in Western blot for detection of ARP1 and ARP3.

| Target gene | Plasmid | 1° antibody | 2° antibody | Detection level |
| --- | --- | --- | --- | --- |
| ARP1-E-tag | pAF100-ARP1 | Mouse monoclonal anti-E-tag antibody (Phadia AB, Uppsala, Sweden, 1 µg/ml) | HRP conjugated goat anti-mouse antibody  (Dako A/S, Glostrup Denmark, 1:1000) | ++ |
| ARP1-E-tag | pAF900-ARP1 | Mouse monoclonal anti-E-tag antibody (Phadia AB, Uppsala, Sweden, 1 µg/ml) | HRP conjugated goat anti-mouse antibody  (Dako A/S, Glostrup Denmark, 1:1000) | ++ |
| ARP1-E-tag | pAF1200 | Mouse monoclonal anti-E-tag antibody (Phadia AB, Uppsala, Sweden, 1 µg/ml) | HRP conjugated goat anti-mouse antibody  (Dako A/S, Glostrup Denmark, 1:1000) | ++ |
| ARP1-E-tag | pAF1300 | Mouse monoclonal anti-E-tag antibody (Phadia AB, Uppsala, Sweden, 1 µg/ml) | HRP conjugated goat anti-mouse antibody  (Dako A/S, Glostrup Denmark, 1:1000) | ++ |
| ARP1-E-tag | pAF1400 | Mouse monoclonal anti-E-tag antibody (Phadia AB, Uppsala, Sweden, 1 µg/ml) | HRP conjugated goat anti-mouse antibody  (Dako A/S, Glostrup Denmark, 1:1000) | ++ |
| ARP3-VSV-tag | pAF900-ARP3(VSV) | Mouse monoclonal anti-VSV-G tag antibody  (Roche, Basel, Switzerland, 1:2500) | HRP conjugated goat anti-mouse antibody  (Dako A/S, Glostrup Denmark, 1:1000) | - |
| ARP3-VSV-tag | pAF900-ARP3(VSV) | Biotinylated polyclonal rabbit anti-VSV-G tag antibody  (Abcam, Cambridge, UK, 1:2000) | HRP conjugated streptavidin (Becton, Dickinson and Company, Franklin Lakes, NJ, USA, 1:1000) | + |
| ARP3-HA-tag | pAF900-ARP3(HA) | Rabbit anti-HA-tag antibody (Sigma-Aldrich, St. Louis, MO, USA, 1:1000) | HRP conjugated swine anti-rabbit antibody (Dako A/S, Glostrup Denmark, 1:1000) | - |
| ARP3-FLAG-tag | pAF900-ARP3(FLAG) | Rabbit anti-FLAG-tag antibody (Sigma-Aldrich, St. Louis, MO, USA, 1:1000) | HRP conjugated swine anti-rabbit antibody (Dako A/S, Glostrup Denmark, 1:1000) | - |
| ARP3-V5-tag | pAF900-ARP3(V5) | Rabbit anti-V5-tag antibody (Sigma-Aldrich, St. Louis, MO, USA, 1:1000) | HRP conjugated swine anti-rabbit antibody (Dako A/S, Glostrup Denmark, 1:1000) | + |
| ARP3-V5-tag | pAF900-ARP3(V5)  pAF1200  pAF1300  pAF1400 | Biotinylated mouse monoclonal anti-V5-tag antibody (AbD Serotec, Kidlington, UK, 1:5000) | HRP conjugated streptavidin (Becton, Dickinson and Company, Franklin Lakes, NJ, USA, 1:1000) | ++ |

“-”: No detection; “+”: Low detection; or “++”: High detection.
