# Supplementary material for: Co-Expression of Anti-Rotavirus Proteins (Llama VHH Antibody Fragments) in Lactobacillus: Development and Functionality of Vectors Containing Two Expression Cassettes in Tandem
Source: PLoS One. 2014 Apr 29;9(4):e96409. doi: 10.1371/journal.pone.0096409 (PMC4004553; doi:10.1371/journal.pone.0096409)
Supplement: Materials and Methods S1 — (DOCX) [file pone.0096409.s003.docx]

**Materials and Methods S1**

**Construction of pAF900 plasmids**

Before construction of the lactobacilli co-expressing ARP1 and ARP3, another tag was selected for fusion to ARP3 in order to detect each antibody fragment separately. The gene encoding the sequence of ARP3, VSV-G-tag, *prtP* and transcription terminator of the *apf* gene was synthesized (Genscript, Piscataway, NJ). During the synthesis, restriction sites were added including *Xho*I and *Bgl*II restriction sites upstream the ARP3 gene, a *Mlu*I between the ARP3 and VSV-G-tag genes, a *Nhe*I after the VSV-G-tag and an *EcoR*I downstream the *apf* terminator (Fig. 1 A). Furthermore, the sequence coding for ARP3, VSV-G-tag and *prtP* was optimized according to the codon usage of *L. paracasei* in order to reduce potential recombination between homologous DNA sequences in the ARP1/ARP3 double expression cassettes.

The synthetic gene was then cloned in the pUC57 plasmid, generating pUC57-ARP3(VSV)-PrtP. In order to fuse the promoter and signal peptide to the synthetic gene, the *apf* promoter sequence and the signal peptide were amplified from pAF900-ARP1 using the primers TR_5_F and TR_6_R (Table S1) and inserted between the *Xho*I and *Bgl*II restriction sites in pUC57-ARP3(VSV)-PrtP generating pUC57-P-SP-ARP3(VSV)-PrtP. The whole expression cassette was subsequently amplified from pUC57-P-SP-ARP3(VSV)-PrtP using TR_5_F and TR_4 primers (Table S1) introducing a *Xho*I uspstream the promoter and *BamH*I, *EcoR*I and *Pvu*I restriction sites downstream of *apf* terminator. The amplicon generated by Taq DNA polymerase reaction was ligated into the pGEM-FT vector linearized by digestion with *Xcm*I [[26](#_ENREF_26)]. The pGEM-FT vector contains an *EcoR*I restriction site upstream the cloned expression cassette. The expression cassette was subsequently released with *EcoR*I and *Pvu*I restriction enzymes and ligated between these restriction enzyme sites in linearized pIAV7 plasmid [[25](#_ENREF_25)] generating pAF900-ARP3(VSV). Subsequently, the VSV-G-tag was replaced with the codon adjusted nucleotide sequences of the V5- (GKPIPNPLLGLDST), HA- (YPYDVPDYA) and FLAG- (DYKDDDDK) tags. Each respective tag encoding gene was amplified from pAF900-ARP3(VSV) plasmid by PCR, using the TR_4 primer in combination with reverse primers encoding each tag (Fw_MluI_FLAG, Fw_MluI_HA or Fw_MluI_V5) (Table S1). The amplicons were digested with *Mlu*I and *Pvu*I restriction sites and ligated in similarly digested pAF900-ARP3(VSV) generating pAF900-ARP3(FLAG), pAF900-ARP3(HA) or pAF900-ARP3(V5) plasmids, respectively (Fig. 1 B).

**Construction of pAF1300 and pAF1400 plasmids**

The plasmid pAF900-ARP3(V5) was digested using *Bgl*II and *Pvu*I restriction enzymes and the fragment containing ARP3, the V5-tag, *prtP* region, non-translated APF C-terminal domain and *apf* terminator region was ligated between the same restriction enzyme sites into pAF1300-VSV and pAF1400-VSV, generating pAF1300 (Fig. 1 C) and pAF1400 (Fig. 1 D), respectively.
